# The future of quantum technologies: superfluorescence from solution-processed, tunable materials

**DOI:** 10.1515/nanoph-2023-0919

**Published:** 2024-02-28

**Authors:** Brendan Russ, Carissa N. Eisler

**Affiliations:** University of California, Los Angeles, Los Angeles, USA

**Keywords:** superfluorescence, superradiance, solution-processed, colloidal nanocrystal

## Abstract

One of the most significant and surprising recent developments in nanocrystal studies was the observation of superfluorescence from a system of self-assembled, colloidal perovskite nanocrystals [G. Rainò, M. A. Becker, M. I. Bodnarchuk, R. F. Mahrt, M. V. Kovalenko, and T. Stöferle, “Superfluorescence from lead halide perovskite quantum dot superlattices,” *Nature*, vol. 563, no. 7733, pp. 671–675, 2018]. Superfluorescence is a quantum-light property in which many dipoles spontaneously synchronize in phase to create a collective, synergistic photon emission with a much faster lifetime. Thus, it is surprising to observe this in more inhomogenous systems as solution-processed and colloidal structures typically suffer from high optical decoherence and non-homogeneous size distributions. Here we outline recent developments in the demonstration of superfluorescence in colloidal and solution-processed systems and explore the chemical and materials science opportunities allowed by such systems. The ability to create bright and tunable superfluorescent sources could enable transformative developments in quantum information applications and advance our understanding of quantum phenomena.

## 1 Introduction

Approaching ever smaller material domains has led to the description and observation of extraordinary optical phenomena. Understanding how light can be quantized has allowed us to understand and observe phenomena such as single photon emission, quantum entanglement, and, the subject of this perspective, superfluorescence [1], [2]. Superfluorescence is a quantum optic phenomenon where a system of initially uncorrelated fluorescing dipoles spontaneously aligns to generate a macroscopic, collective dipole that emits a burst of coherent photons at a fast rate [3]. This effect is very similar to superradiance, but unlike superradiance, the system does not have an initial macroscopic dipole moment; the superfluorescence is created by the spontaneous generation of a macroscopic aligned dipole. A simplified analogy of this effect would be akin to a stadium of people randomly cheering until they all spontaneously create one collective chant at the same time. Collective phenomena like this have been observed in large scale, classical systems: animals that travel in groups, such as flocks of birds, will move as a collective unit without a group leader or external stimulus because of the spontaneous interaction with their neighbors [4]. However, superfluorescence is a distinctly quantum cooperative effect because it results from the tiny fluctuations, or noise, in the spontaneous emission of the individual dipoles [5], [6]. This quantum property allows for unique and desirable photophysics.

The study and experimental demonstration of superfluorescence is important from both a fundamental and an applied perspective. Because superfluorescence is a quantum-based, collective interaction from many individual dipoles, superfluorescence could be an approach to experimentally study the many-body physics problem in systems that undergoes a macroscopic phase transition (i.e. from being uncorrelated to being correlated) [7], [8]. Studying these properties could hopefully elucidate the complex quantum mechanical behavior that creates entanglement [9]. Optimizing materials for superfluorescence could also enable huge advances in optoelectronics. Superfluorescence and superradiance could be exploited in the development of an ultranarrow, coherent light source laser [10], [11]. Further, the next generation of information processing, analysis, and transmission will rely on quantum mechanics. In order to meet our ever increasing computation demands, we can use coherent quantum systems that significantly speed up complex calculations and improve the security of sharing information [2], [12].

In order to realize these fundamental platforms and new applications, we need very bright sources of entangled photons that come from scalable processes and operate under achievable standard conditions. Historically, this has been difficult to experimentally realize, but recent work has shown exciting developments in coherent and cooperative light emission from solution-processed materials [10], [13], [14], [15], [16], [17], [18], [19], [20]. Here, we review the recent developments in this area to provide (1) context of what superfluorescence and collective photoluminescence emission is, (2) historical perspective of what materials system this phenomenon has been observed in, and (3) guiding principles as to why some colloidal or solution-processed systems have demonstrated this phenomenon (e.g. perovskites nanocrystals and thin films) and how we can take advantage of this to make significant advances in future quantum devices.

## 2 Overview of superfluorescence theory

Cooperative emission from a system of identical two-level atoms was first suggested by R. H. Dicke in 1954 using predominantly classical methods [3]. Dubbed superradiance, Dicke predicted that *N* identical atoms all initially in the excited state and with an initial macroscopic dipole could emit in a coherent burst, owing to the interaction between each dipole and the overall electromagnetic field. Superfluorescence, which differs from superradiance in that there is no initial macroscopic dipole aligning the atoms, was first described by Joseph H. Eberly and Nicholas E. Rehler in 1969 for volumes much smaller than the emission wavelength in terms of a single laser model [21], [22] and then described through quantum-mechanics by R. Bonifacio and L. A. Lugiato in 1975 and 1976 [5], [6]. In the quantum mechanics treatment, the propagation effects of light through the system are neglected, allowing some quantitative analysis for simple cases. The key result from this treatment is that the superfluorescent emission behaves as a pendulum controlled by the various timescales of the different electronic and photonic processes in the sample. This work was later extended by Friedberg, Hartman, and Manassah to include more accurate dipole-dipole coupling, called Van der Waals dephasing [23]. Gross and Haroche unified much of the theory relating superfluorescence and superradiance in 1982 [24]. This work also extended the theoretical methods to include a large variety of additional effects present in experimental conditions but largely ignored or neglected by theoreticians previously, such as multi-level superfluorescent systems [24].

The quantum mechanics treatment of this system allows us to predict the conditions under which superfluorescence can occur [5], [6]. The emission follows pendulum-like dynamics dependent on the following time scales of the sample: *τ*_*R*_, the length of time of the superfluorescent radiation; *τ*_*D*_, the reduced delay time (also frequently described as the alignment time); *τ*_*c*_, the characteristic time by which the atoms and fields exchange energy; $T_{2}^{*}$ , the dephasing time, which is also taken as the smallest atomic relaxation time; and *τ*_*p*_, which is the characteristic time a photon exists in the active volume. Under the condition that the peak emission wavelength is much smaller than the dimensions of the “pencil-shaped” rod geometry, superfluorescence arises when the emitted photons leave the volume much faster than the superfluorescence lifetime (*τ*_*R*_) and the dephasing time ( $T_{2}^{*}$ ). Intuitively this means that a system must emit collective photons faster than these collective states can dephase, necessitating that relaxation events and trap states are minimized.

**Figure 1:**
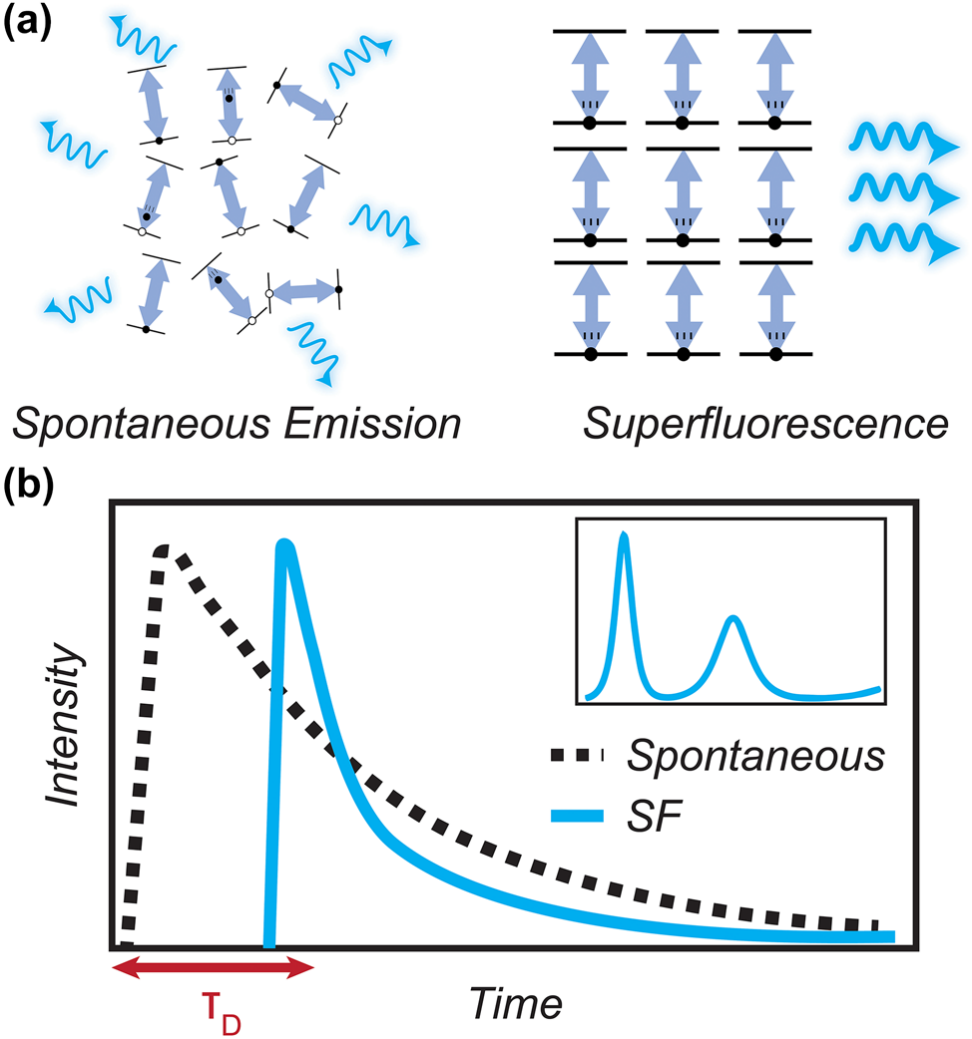
Characteristic properties of superfluorescent emission. (a) Schematic of spontaneous emission (left) and cooperative emission (right). Dipoles emitting spontaneously are not aligned and emit incoherently at different times. Dipoles emitting cooperatively are aligned and emit coherently at similar times. (b) Schematic of time-resolved photoluminescence characteristic of spontaneous emission and superfluorescence. Notably, superfluorescence emits at a faster time scale and with a characteristic time delay (*τ*_*D*_). Inset: example of Burnham-Chiao ringing (subsequent peaks in time-resolved photoluminescence) can appear depending on excitation power and sample geometry.

The characteristic properties of superfluorescent emission arise from the coherent coupling between *N* emitters due to their shared interaction with the electromagnetic field, as shown in Figure 1. Compared to spontaneous emission, superfluorescence emits in a burst faster than spontaneous emission by a factor of *N* (Figure 1b). That is, the *τ*_*R*_ ∝ *τ*_SE_/*N*, where *τ*_SF_ is the superfluorescent emission lifetime and *τ*_SE_ is the spontaneous emission lifetime. The intensity of the superfluorescence peak scales as *I* ∝ *N* ^2^ whereas spontaneous emission scales as *I* ∝ *N*. Another characteristic that sets superfluorescence apart from spontaneous emission is that superfluorescence has a characteristic time delay between excitation and emission, as shown in Figure 1b, which arises due to the time needed for the dipoles to spontaneously align in phase. Superfluorescent emission frequently exhibits Burnham-Chiao ringing, an oscillation in the time-resolved photoluminescence, due to reabsorption and stimulated emission in a sample of emitters, usually when the photons exist in the active volume of superfluorescent emission on time scales comparable to the superfluorescence lifetime. This is what gives rise to the decaying oscillatory peaks in Figure 1b. Because the dipoles are spontaneously aligned, the final key characteristic is that the superfluorescent emission is coherent instead of being incoherent emission from *N* spontaneous emission sources.

In trying to observe superfluorescence, one must be careful to distinguish the superfluorescence phenomenon from other similar effects. Amplified stimulated emission competes with superfluorescent emission under certain conditions; the key difference is that superfluorescent emission always scales with *I* ∝ *N* ^2^ while amplified stimulated emission can include other nonlinear intensity scaling power laws. Realistic samples larger than the superfluorescent wavelength will likely include some amount of both superfluorescence and amplified stimulated emission. Superradiance is largely the same as superfluorescence, except that superradiance arises when there is an existing initial macroscopic dipole alignment dictated by the experiment. To differentiate between superfluorescence and superradiation requires measurement of the dipole alignment: superfluorescence will align to random dipoles while superradiation will always align to the induced dipole. This differentiation can also be made by measuring the polarization of the emission burst: superradiant emission will be polarized in accordance with the induced dipole, while superfluorescent emission will be polarized randomly from shot to shot [25]. Additionally, most experimental samples will include a variety of other factors not included in the ideal theory for superfluorescence. These can manifest as redshifting in the emission wavelength, varying delay times, diffraction in the emission burst, washing out of the ringing effects, the appearance of superimposed waves, inconsistency in superfluorescent occurrences, and sharp cutoffs to the superfluorescent peaks. Some of the origins of these variations are explored in much more detail in literature [24].

## 3 The surprising observation of superfluorescence in inhomogeneous, solution-processed systems

Superradiance was first experimentally confirmed in 1973 in HF gas, where coherent, collective emission was generated via the synchronization of transition between two rotational states [26]. Since then, superradiance and superfluorescence has predominantly been observed in highly homogenous systems, such as small molecular and atomic gases and atomic impurities in solid crystals [27], [28], [29], [30], [31], [32], [33], [34]. While studying highly defined states decreases the dephasing, it does also limit the applications and manufacturing scalability. Thus an exciting development has been the demonstration of cooperative emission in more inhomogeneous systems such as semiconductor quantum dots ensembles [35], [36] and quantum wells [8]. Even though these systems have higher decoherence than previously demonstrated atomic systems, superfluorescence is indeed possible when there is either the presence of homogenous dipoles (such as specific atomic states) within the inhomogenous system or significant light-matter coupling in the inhomogenous material leading to much faster radiative lifetimes [37]. Further, these demonstrations were limited to extremely low cryogenic temperatures or under high magnetic fields [8].

**Figure 2:**
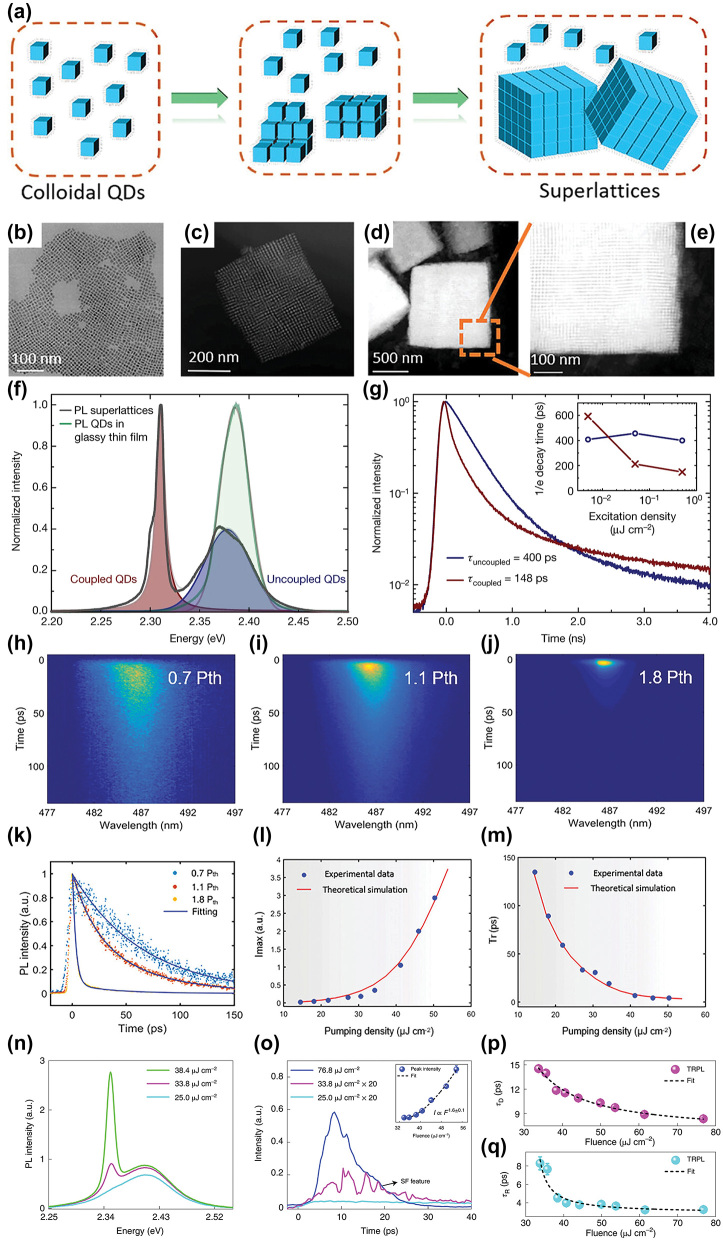
Superfluorescence from solution-processed, scalable materials. (a)–(j) Superfluorescence demonstrated in perovskite nanocrystal superlattices. (a)–(e) Schematic and images of QD assembly to superlattice from [18], (b)–(e) show TEM images of CsPbBr_2_Cl nanocrystals and their assembly into superlattices at varying magnification. (f)–(g) Demonstration of superfluorescence from CsPbBr_3_ nanocrystal superlattice from [10]. (f) Characteristic redshift and narrowing peak of coupled (assembled into a superlattice) CsPbBr_3_ QDs compared to uncoupled (film of QDs). (g) Time-resolved photoluminescence of an unassembled film of nanocrystals (blue) versus an assembled and correlated superlattice (red). The faster excitation time in the superlattice assembly compared to the film assembly is characteristic of superfluorescence. (h)–(m) Demonstration of superfluorescence from CsPbBr_2_Cl nanocrystal superlattice [18]. Streak camera images showing the time and wavelength dependence of superfluorescent emission under varying power densities. 1.0 Pth indicates the power required to reach cavity-enhanced superfluorescence, which decreases the emission time and narrows the emission peak. (k) Time-resolved photoluminescence showing the characteristic decay times for the data recorded in (h)–(j). (l) Fluorescence intensity as a function of applied pumping density. The exponential increase indicates increased cooperative emission effects as the pumping density increases. (m) Characteristic radiation time as a function of pumping density. The decrease in excitation time as pumping density increases corresponds to increased cooperative emission effects. (n)–(q) Demonstration of superfluorescence from (PEA)CsPbBr_3_ thin films [14]. Photoluminescence intensity is plotted versus energy (n) and time (o). Note the characteristic Burnham-Chiao ringing in the time-resolved photoluminescence (o) at higher fluences. (p) and (q) Show the delay time and real width, respectively, extracted from time-resolved photoluminescence measurements at different excitation fluences. (a–e) Reproduced with Permission [18]. (f–g) Reproduced with Permission [10]. Copyright 2018, Springer Nature. (h–m). Reproduced with Permission [18]. (n–q) Reproduced with Permission [14]. Copyright 2022, Springer Nature.

While these solid-state systems studies show promise for practical applications, these cases were still limited to pristine materials fabricated via more complex vapor deposition techniques and extreme operating conditions. Thus, one of the most significant and most surprising developments recently was the observation of superfluorescence from solution-processed, scalable materials (Figure 2). In 2018, Rainò et al. demonstrated superfluorescence from colloidally-synthesized perovskite (CsPbX_3_ where X = Br, Cl) nanocrystals that self-assembled into micron-sized superlattices [10]. These superlattices showed the typical signatures of superfluorescence at low temperatures (6 K): fast, narrowband, and coherent photoluminescence with the signature time delay and Burnham-Chiao ringing at high laser fluences (Figure 2g). The superfluorescence emission of the superlattice was redshifted by 64 ± 6 meV relative to the emission of the individual, uncorrleated nanocrystals (Figure 2f), but this could be explained by the thermal decoherence and structural disorder of the superlattice [38]. It was determined that the structure of the superlattice drove the superfluorescence: the coherence lifetime was more than 3.5× greater for the perovskite superlattice than the uncoupled, unassembled perovskite nanocrystals. This has led to expanded studies on the superfluorescence from these perovskite nanocrystal superlattices [10], [13], [17], [18], [19], [20], [39]. Notably, Zhou et al. observed superfluorescence from these perovskite nanocrystal superlattices at even higher temperatures (77 K) because of the formation of extremely uniform superlattices (Figure 2a–e) [13]. These superlattices act like optical cavities, reducing the photoluminescence lifetime by more than 3000× when compared to the individual nanocrystals (Figure 2h–m). Finally, room-temperature, upconverted superfluorescence was demonstrated in Nd^3+^-ion-doped upconverting nanoparticles [16]. Because the superfluorescence comes from the lanthanide dopants (Nd^3+^) and not the nanocrystal, these act as a sort of hybrid homogenous-inhomogenous system, where the superfluorescence originates from the highly defined, less-disturbed 4f electron transitions of the lanthanide doped ions with very small inter-emitter distances [16], [37]. Thus superfluorescence can be observed from even single nanocrystals in this system. All of these are very exciting developments, demonstrating the potential for quantum light sources from scalable and small-scale nanomaterials.

The development of high quality, solution-processed materials has only accelerated in the last few years, and this has led to the exciting observation of superfluorescence at higher temperatures in solution-processed inhomogenous systems, namely perovskite thin films [14], [15]. Notably, Biliroglu et al. demonstrated room-temperature superfluorescent emission in hybrid organic-inorganic ((PEA)CsPbBr_3_) perovskite thin films [14]. This was a very important development as this demonstrated superfluorescent emission in an inhomogenous, solution-processed material at elevated temperatures. They attributed the desirable photophysical properties to what they called the *vibration-isolation postulate*, wherein the formation of large polarons, or hybrid exciton-phonon quasiparticles, created the necessary isolation between the CsPbBr_3_ dipoles and phonons to enable superfluorescence [14]. These thin films had a mixture of 2D and 3D (quantum confined and unconfined) domains arising from the mix of bulky phenylethyl ammonium (PEA) cations that insulate and isolate PbBr_6_ octrahedra (2D) and smaller cesium (Cs) cations that create closer packed, bulk-like CsPbBr_3_ regions (3D). They postulated that these regions of confinement created large polarons that prevented exciton dephasing, leading to effective superfluorescence. The implications of these recent studies are very exciting: even in the disorder of solution-processed, scalable materials, extraordinary optical phenomena can be observed at room temperature with careful materials design.

Finally, this recent work has also brought to light the importance of accurately identifying superfluorescence – especially in perovskite superlattices. Baranov et al. demonstrated redshifted emission bursts with Burham-Chiao ringing in bulk-like CsPbBr_3_ particles without the characteristic coherence present in superfluorescent emission [40] and Qiang et al. observed superbunching in cascade emission in mesoscopic CsPbBr_3_ nanostructures [41]. As discussed in Section 2, many of the properties of superfluorescence are shared among other photoluminescence phenomenon. Thus, future studies should take great care to prove the observation of superfluorescence through the numerous criteria.

## 4 How can we realize emergent quantum technologies in these scalable, solution-processed systems?

Despite the larger size- and structure-inhomogeneities of these solution-processed materials, superfluorescence has been successfully demonstrated in colloidal nanocrystals and solution-processed thin films. The doping of highly defined states, such as rare-earth ions, within solution-processed materials allows for a pathway to superfluorescence from the homogenous atomic states within an inhomogenous colloidal material [14]. Here, we will focus on the development of fully inhomogenous material systems, namely perovskite nanocrystal superlattices and thin films. This begs the question: what makes this material system special? Perovskites can seemingly overcome energetic inhomogeneities that would otherwise preclude them from the strong emitter coupling required for superfluorescence. The perovskite crystal’s soft lattice lends high structural defect tolerance, which when combined with the unusually emissive lowest exciton energy level enables strong energetic homogeneity in an otherwise inhomogeneous system [42], [43]. Additionally, surface engineering and size selection of perovskite nanocrystals has shown significant emission peak narrowing, further expanding the degree to which perovskites overcome the energetic impact of inhomogeneities [44], [45]. Recent work demonstrating superfluorescence in perovskite nanocrystal superlattices has shown very narrow photoluminescence emission: the full-widths at half maximum (FWHM) range from roughly 30–50 meV for the uncoupled perovskite nanocrystals [10], [17], [18]. The fast radiative lifetimes in these materials also play a strong role. In nanocrystal superlattices, radiative lifetimes are decreased because the nanocrystals can act as an ensemble and couple into shared states [36], [46]. Additionally, it has been shown that colloidal superlattices have collective vibrational states through the linking of the surface ligands [47], which could be an important mechanism as polaron formation has been hypothesized as an exciton dephasing mechanism in other work [14]. However, it is notable the *perovskite* nanocrystal system specifically has demonstrated superfluorescence over other colloidal nanocrystal systems. Recent work into perovskite nanocrystals has shown that these materials inherently have very fast radiative lifetimes and long exciton dephasing times that are comparable to other known “high coherence” systems such as atomic defects in diamond and epitaxial III–V quantum dots [48], [49]. This is highly unique for this material system as most other high efficiency colloidal nanocrystal systems, such as CdSe/ZnS and PbS, often have much faster decoherence times compared to their radiative lifetimes by many orders of magnitude [50], [51]. These favorable timescales have been attributed to the high oscillator strength of the material and to the absence of lower energy dark states [48] and are the reason that other extraordinary optical phenomena, such as coherent single photon emission, has been observed in these nanocrystals [52], [53]. These desirable lifetimes are also seen in thin films, and in particular from either 2D or mixed phase 2D–3D systems where there is increased exciton binding from the type-1 quantum wells [54], [55]. There are ongoing questions into how the surface and grain boundaries play a role in coherence, but these thin films have been able to achieve higher dephasing times leading to applications such as strong spin-orbit coupling [56], [57].

**Figure 3:**
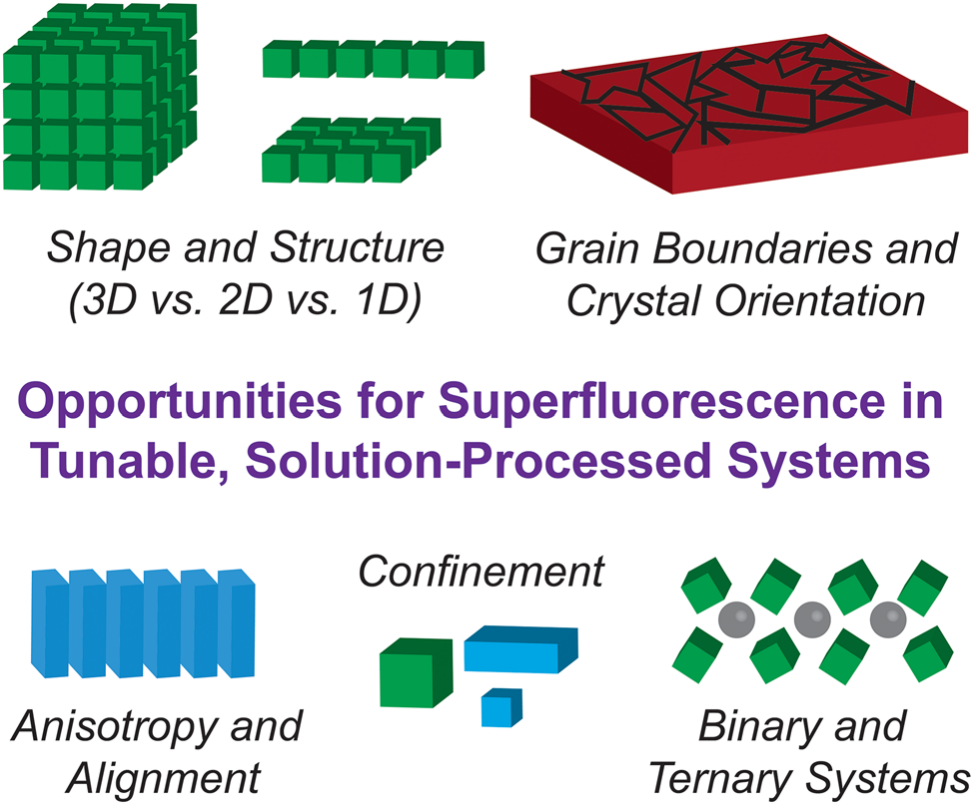
Schematic of some opportunities for advanced studies in superfluorescence afforded by tunable, solution-processed materials systems. From top left going counter-clockwise: changing the superlattice structure [64], exploring the effect of grain boundaries and crystal orientation in thin films, creating binary and ternary superlattices from multiple nanocrystals [20], changing the size and confinement of particles and films [18], [38], and using anisotropy and alignment of particles and film phases.

One of the greatest advantages of this materials system is its facile tunability. By tuning the chemical and structural parameters of this material system, we can gain more fundamental insight into what allows these materials to demonstrate these properties while simultaneously producing a suite of quantum emitters of varying colors and sizes. Some of these qualities are summarized in Figure 3. The materials highlighted in this perspective have mostly been lead halide perovskites (APbX_3_) where A is a positively charged cation and X is a negatively charged halide atom. Changing the halide atom (Br, Cl, I) and the cation (Cs, PEA, etc.) shifts the band edge of the material [58], [59], [60]. Further, the size of the cation can also change the local confinement as larger organic cations (e.g. PEA) create quantum wells for the lead-halide octahedra [14], [59]. Further, the structuring and ordering of both the thin films and the nanocrystal superlattices have strong effects on the optoelectronic properties and the quantum properties. For thin films, the photophysics of the perovskite material is highly tied to the dimension, crystal phase, confinement, and grain boundaries (size and orientation) [61], [62]. Thus the orientation and strain profile of these confined regions could be manipulated to offer optical anisotropy and perhaps enhanced collective emission [63]. For nanocrystal superlattices, theoretical studies have shown that either decreasing the size of the perovskite nanocrystal or changing the nanocrystal to more anisotropic shapes can increase the coupling between the individual emitters and provide a sufficient pathway to increased superfluorescence [38]. Additionally, depending on the number of emitters coupled (*N*), different superlattice dimensionalities (i.e. whether the superlattice itself is 3D or 2D) will be more optimal for observing superradiance and superfluorescence. For example, for less than 1000 emitters coupled, 2D superlattices (i.e. monolayers) could be more robust to static and thermal disorder [64]. Finally, the structure of the superlattice will play a significant role. As with the films, the strain profile affects the carrier dynamics of the system, and this can change over time or over varying experimental conditions [40]. Also, superlattices are not restricted to one material or shape and can be made with multiple building blocks to create more complex crystal structures and shapes [65]. Cherniukh et al. recently demonstrated superfluorescence from perovskite nanocrystals within a highly organized ternary superlattice composed of CsPbBr_3_, Fe_3_O_4_ or NaGdF_4_, and PbS nanocrystals [20], showing immense promise for studies in superfluorescence where the environment, spacing and orientation of the emitters can be independently tuned.

Like many quantum phenomena, the observation and application of superfluorescence is no longer just a condensed matter physics challenge but also a chemistry and materials engineering challenge. By observing of this effect in perovskite nanocrystal superlattices and thin films, we now have a highly tunable material platform where band structure, surface chemistry, materials strain, and more can be correlated to extraordinary optical behavior. This is an immensely exciting opportunity as we believe further study and exploration of the effects afforded by this materials system will allow us fundamental insights into quantum behavior and novel technologies for next generation computing and metrology.
